# Plasma level of lipocalin 2 is increased in neovascular age-related macular degeneration patients, particularly those with macular fibrosis

**DOI:** 10.1186/s12979-020-00205-w

**Published:** 2020-11-14

**Authors:** Mei Chen, Nan Yang, Judith Lechner, Levente Toth, Ruth Hogg, Giuliana Silvestri, Usha Chakravarthy, Heping Xu

**Affiliations:** 1 Centre for Experimental Medicine, The Wellcome-Wolfson Institute for Experimental Medicine, School of Medicine, Dentistry & Biomedical Science, Queen’s University Belfast, 97 Lisburn Road, Belfast, BT9 7BL UK; 2 Centre for Public Health, School of Medicine, Dentistry & Biomedical Science, Queen’s University Belfast, 97 Lisburn Road, Belfast, BT9 7BL UK; 3 Belfast Health and Social Care Trust, Belfast, BT12 6BA UK

**Keywords:** Age-related macular degeneration, Inflammation, Neutrophils, Lipocalin 2, Matrix metalloproteinase, Macular fibrosis, Plasma

## Abstract

**Background:**

Previously, we and others have reported higher populations of circulating neutrophils in patients with neovascular age-related macular degeneration (nAMD). Neutrophil gelatinase-associated lipocalin (NGAL, also known as lipocalin-2, LCN2), an important innate immune mediator, is known to be critically involved in sterile inflammation-mediated organ failure, fibrosis, cancer progression and retinal degeneration. This study investigated the plasma levels of LCN2, matrix metalloproteinase 9 (MMP9) and LCN2/MMP9 complex in different types of nAMD and examined whether the levels were related to patients’ responsiveness to anti-VEGF therapy.

**Results:**

One hundred and seventy-four nAMD patients, including 108 with choroidal neovascularisation (CNV), 32 with retinal angiomatous proliferation (RAP), 23 with polypoidal choroidal vasculopathy (PCV) and 11 unclassified patients, and 43 healthy controls were recruited to this case-control study. Fifty-eight nAMD patients had macular fibrosis and 110 patients did not. Out of the 174 nAMD patients, 80 patients responded completely, 90 responded partially, and 4 did not respond to the anti-VEGF therapy. The plasma levels of LCN2 in nAMD patients (181.46 ± 73.62 ng/ml) was significantly higher than that in healthy controls (152.24 ± 49.55 ng/ml, *P* = 0.047). However, the difference disappeared after adjusting for age. A positive correlation between plasma level of LCN2 and age was observed in nAMD patients (*r* = 0.29, *P* = 0.0002) but not in healthy controls. The plasma level of LCN2 was also positively correlated with circulating neutrophils in nAMD patients (*r* = 0.34, *p* = 0.0007) but not in healthy controls (*r* = 0.057, *p* = 0.77). No correlation was observed between age and circulating neutrophils. Further analysis of nAMD subtypes uncovered a significantly higher level of LCN2 in patients with macular fibrosis even after adjusting for age. No relationship was observed between plasma levels of LCN2 and patients’ responsiveness to anti-VEGF therapy. The plasma levels of MMP9 and LCN2/MMP9 complex were comparable between nAMD and controls.

**Conclusions:**

Our results suggest that higher plasma levels of LCN2 in nAMD are related to ageing and increased population of circulating neutrophils. Our results also suggest that higher levels of LCN2 may increase the risk of macular fibrosis in nAMD.

## Background

Age-related macular degeneration (AMD) is the progressive degeneration and loss of function of the central part of the back of the eye, the macula, resulting from old age. AMD is the leading cause of blindness in the elderly in developed countries [1]. There are two advanced forms of AMD, geographic atrophy (GA) and neovascular AMD (nAMD) [2]. nAMD can be further classified into three subtypes, (a) choroidal neovascularisation (CNV), characterised by infiltration of abnormal blood vessels from the choroid into the sub-retinal pigment epithelial (RPE) or sub-retinal space [2]; (b) retinal angiomatous proliferation (RAP) [3], the de novo growth of new blood vessels from retinal vessels that can fuse with CNV; (c) polypoidal choroidal vasculopathy (PCV), sub-RPE infiltration of choroid-derived polypoidal lesions [4]. nAMD account for 80% of AMD-mediated visual loss [5] and is currently treated with intravitreal injection of vascular endothelial growth factor (VEGF) inhibitors. Although the therapy can stabilise or even improve visual function [6], around a third of patients do not respond to the therapy [7]. Furthermore, approximately 50% of treated eyes may develop macular fibrosis and those patients may ultimately lose vision [8]. Currently, there is no treatment for GA. Pathogenesis of AMD is not fully understood although inflammation is believed to play an important role [9, 10].

Exactly how inflammation leads to macular damage in AMD remains poorly defined. Increased systemic and local inflammatory responses, including complement activation [11–15], immune cell alteration [16–19] and cytokine production [20–24] have been observed in AMD. Previously, we and others have reported increased circulating neutrophils in nAMD patients [19, 25, 26]. Neutrophils are the major type of innate immune cells making up 40–70% circulating leukocytes. Apart from their anti-microbial functions, neutrophils are also known to participate in various non-infectious sterile inflammations, for example by releasing inflammatory mediators and neutrophil extracellular traps (NETs). The neutrophil gelatinase-associated lipocalin (NGAL, also known as lipocalin-2, LCN2) is constitutively released to the bloodstream by neutrophils under normal physiological conditions [27]. LCN2 binds to the siderophores of bacteria limiting their growth [27]. LCN2 also plays a critical role in iron homeostasis [28], in stabilising matrix metalloproteinase 9 (MMP9) [29], and higher levels of MMP9 is known to be involved in AMD [30]. The production of LCN2 is often increased in disease conditions, and it is known to be involved in sterile inflammation, wound-healing and tissue remodelling, fibrosis [31–34], kidney failure [35, 36], neurodegeneration [37, 38] and cancer progression [39]. Importantly, recent studies have shown that LCN2 actively participates in retinal inflammation and contributes to the development of early and dry AMD [40].

We hypothesized that higher circulating levels of neutrophils may contribute to nAMD through the release of LCN2. LCN2 may participate in nAMD development by promoting dysregulated macular inflammation or stabilizing MMP9. This study aimed to understand if the plasma levels of LCN2, MMP9 and LCN2/MMP9 complex are increased in nAMD patients and their relationship with circulating neutrophils. Here, we showed that the plasma level of LCN2, but not MMP9 or LCN2/MMP9 complex, was significantly increased in nAMD patients, particular those with macular fibrosis. We further found that the plasma levels of LCN2 positively correlated to age and circulating neutrophils in nAMD but not controls. Our results suggest that LCN2 may play a role in macular fibrosis secondary to nAMD.

## Results

### Demographic and clinical characteristics of participants

A total of 174 nAMD patients and 43 healthy controls were recruited to this study. Demographic analysis showed no difference between the two groups in gender distribution, history of hypertension and cardiovascular disease, history of diabetes and family history of AMD (Table 1). There was also no difference in smoking status, body mass index (BMI), and psychiatry drug intake between nAMD patients and healthy controls. However, the average age when blood was taken, the number of participants who took vitamin supplements or low-dose aspirin were significantly higher in nAMD group compared with those in controls (Table 1). Since vitamin supplementation is usually advised to AMD patients, the higher number of people taking vitamin supplements in the nAMD group is likely the consequence of the disease, rather than a potential contributor to the disease. Therefore, this was not included in the subsequent analysis of confounders.

**Table 1:** Demographic and clinical characteristics of nAMD patients and controls

	All (*N* = 217)	Control (*N* = 43)	nAMD (*N* = 174)	*P* value
Age, mean (range)	77.3 (53–93)	73.4 (58–92)	78.3 (53–93)	**0.001**^**a**^
Female, number (%)	110 (51)	19 (44)	91 (52)	0.341^b^
Family history of AMD, number (%)	46 (21)	6 (14)	40 (23)	0.210^b^
History of hypertension, number (%)	133 (62)	23 (53)	110 (63)	0.312^b^
History of diabetes, number (%)	28 (13)	2 (5)	26 (15)	0.078^b^
History of cardiovascular disease, number (%)	57 (26)	9 (21)	48 (28)	0.416^b^
Smoking status				0.558^b^
Non-smoker, number (%)	90 (42)	20 (47)	70 (40)	
Former smoker, number (%)	109 (51)	20 (47)	89 (51)	
Current smoker, number (%)	17 (8)	2 (5)	15 (9)	
Body Mass Index (Mean ± SD)	26.0 ± 4.4	26.1 ± 5.1	26.0 ± 4.2	0.967^c^
Taking Cardiovascular Drugs, number (%)	156 (72)	28 (65)	128 (74)	0.370^b^
Taking Vitamins, number (%)	49 (23)	3 (7)	46 (26)	**0.007**^**b**^
Takin low-dose aspirin, number (%)	73 (34)	5 (12)	68 (39)	**0.001**^**b**^

^a^Mann Whitney U test

^b^Pearson’s chi-square test

^c^Independent sample t-test

### Plasma LCN2, MMP9 and MMP9/LCN2 in nAMD patients

**Fig. 1:**
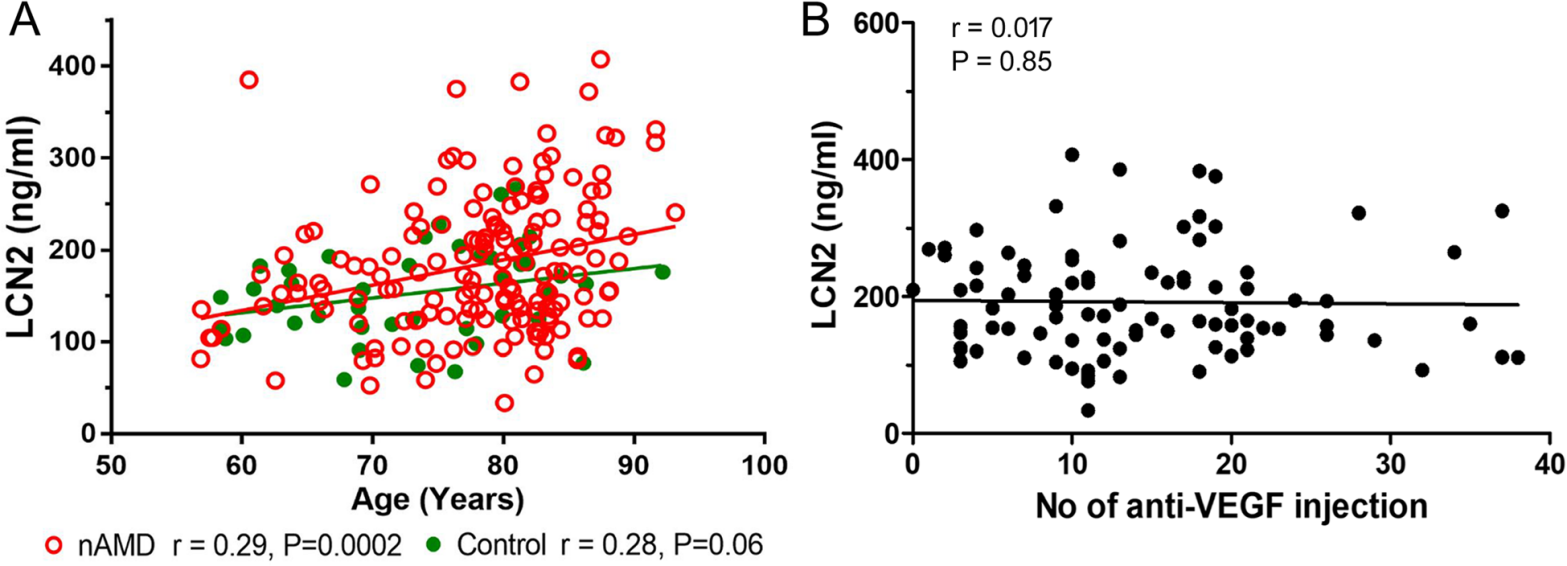
Correlations between plasma levels of LCN2, age, and the number of intravitreal anti-VEGF injections. **a** Correlation between the plasma levels of LCN2 and age in healthy controls (*n* = 43, green) and nAMD (*n* = 168, red) by Pearson’s correlation. **b** Correlation between the plasma levels of LCN2 and the number of anti-VEGF injections at the time of blood sample collection. *N* = 90, Pearson’s correlation

The plasma level of LCN2 in nAMD patients was 181.46 ± 73.62 ng/ml, significantly higher than that in healthy controls (152.24 ± 49.55 ng/ml, *P* = 0.047, independent samples test, Table 2). However, the difference became insignificant after correcting for age or taking low-dose aspirin (*p* = 0.259 and 0.602, respectively, Table 2). A linear regression study uncovered a positive correlation between age and the plasma levels of LCN2 in nAMD patients (*r* = 0.29, *p* = 0.0002, Fig. 1a, red line and red dots). No statistically significant correlation (*r* = 0.28, *p* = 0.06. Figure 1a, green line and green dots) was observed in healthy controls. Our results suggest that plasma levels of LCN2 were positively affected by age, particularly in nAMD patients. No correlation was observed between the plasma levels of LCN2 and the number of intravitreal Lucentis injection in nAMD patients (Fig. 1b).

**Table 2:** Plasma levels of LCN2, MMP9 and MMP9/LCN2 complex in nAMD and controls

Variable (ng/ml)	Control (Mean ± SD)	nAMD (Mean ± SD)	Univariate analysis (*p* value)	Corrected by age (*p* value)	Corrected by taking aspirin (*p* value)	Corrected by taking aspirin and age
	*N* = 43	*N* = 168				
LCN2	153.24 ± 49.55	181.46 ± 73.62	0.047^a^	0.259^b^	0.602^c^	0.396^d^
	*N* = 43	*N* = 167				
MMP9	124.16 ± 63.12	116.36 ± 68.45	0.274^a^			
	*N* = 43	*N* = 167				
MMP9/LCN2	188.38 ± 45.12	179.16 ± 58.11	0.335^a^			

^a^Independent samples t test

^b^Univariate analysis corrected for age

^c^Univariate analysis corrected for taking aspirin

^d^Univariate analysis corrected for age and taking aspirin

There was no difference in the plasma levels of MMP9 and LCN2/MMP9 complex between nAMD patients and healthy controls (Table 2).

**Fig. 2:**
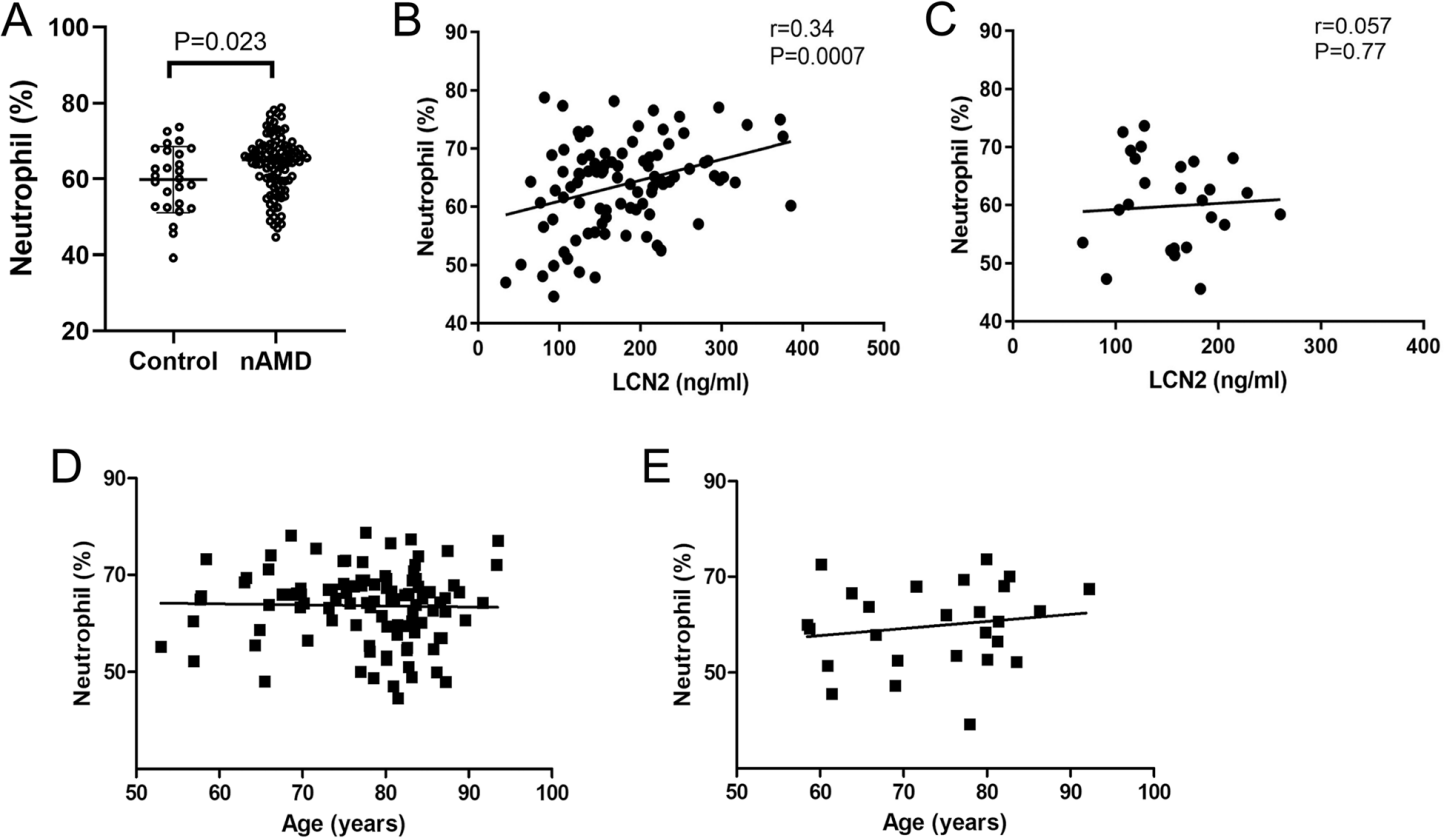
Correlations between circulating neutrophils, plasma levels of LCN2 and age. Neutrophils were identified by flow cytometry of peripheral blood as CD11b ^+^ CD16 ^+^ HLA-DR^-^, and plasma levels of LCN2 were measured by ELISA. **a** Neutrophils in healthy controls and nAMD. Unpaired t-test. **b, c** Correlations between the plasma levels of LCN2 and circulating neutrophils in nAMD (**b**) and healthy controls (**c**). **d, e** Correlations between circulating neutrophils and age in nAMD (**d**) and healthy controls (**e**). nAMD: *n* = 71, Healthy control: *n* = 26. Pearson’s correlation

We previously observed a higher population of circulating neutrophils in nAMD patients [19]. The result was confirmed in this cohort of participants (Fig. 2a). To understand if higher numbers of circulating neutrophils contribute to increased LCN2, a correlation analysis was conducted in the participants whose neutrophil data were collected in our study [19]. As expected, the population of circulating neutrophils was positively correlated with plasma levels of LCN2 in nAMD patients (*r* = 0.34, *p* = 0.0007, Fig. 2b) but not in healthy controls (*r* = 0.057, *p* = 0.77, Fig. 2c). Interestingly, no correlation was observed between age and circulating neutrophils in both nAMD patients (Fig. 2d) and controls (Fig. 2e), suggesting that ageing and circulating neutrophils contribute independently to the increased plasma levels of LCN2 in nAMD patients.

### LCN2 in nAMD subgroups

Out of the 174 nAMD participants, 108 had CNV, 32 with RAP, 23 with PCV and 11 were unknown. One-way ANOVA analysis did not reveal any significant difference in the plasma levels of LCN2, MMP9 or MMP9/LCN2 complex among the control, CNV, RAP and PVC groups (*p* = 0.108, 0.301 and 0.370 respectively, Table 3). Univariate analysis showed that the plasma levels of LCN2 in CNV subgroup were higher than those of control group (*p* = 0.006, Table 3), however, the difference became insignificant after adjusting for age (*p* = 0.064).

**Table 3:** Plasma levels of LCN2, MMP9, and MMP9/LCN2 complex in different types of nAMD patients and controls

Variable	Control (*N* = 43, Mean ± SD)	CNV (*N* = 108, Mean ± SD)	RAP (*N* = 32, Mean ± SD)	PCV (*N* = 23, Mean ± SD)	*p* value
Age	73.4 ± 8.9	78.9 ± 7.8^†^	80.7 ± 8.7^†^	71.6 ± 8.9	
LCN2 (ng/ml)	153.24 ± 49.55	186.60 ± 71.57^*^	176.08 ± 82.70	168.36 ± 70.68	0.108^a^
MMP9 (ng/ml)	124.16 ± 63.12	114.42 ± 58.93	111.18 ± 96.94	126.40 ± 65.09	0.301^a^
MMP9/LCN2 (ng/ml)	188.38 ± 45.12	176.51 ± 53.35	181.69 ± 66.07	196.29 ± 66.05	0.370^b^

*CNV* choroidal neovascularisation, *RAP* retinal angiomatous proliferation, *PCV* polypoidal choroidal vasculopathy

^†^*P* < 0.05 compared to controls

^*^P = 0.006 compared to controls in univariate analysis

^a^Kruskal-Wallis one-way analysis of variance on log transformed data

^b^One-way ANOVA

### LCN2 in nAMD patients with or without macular fibrosis

**Fig. 3:**
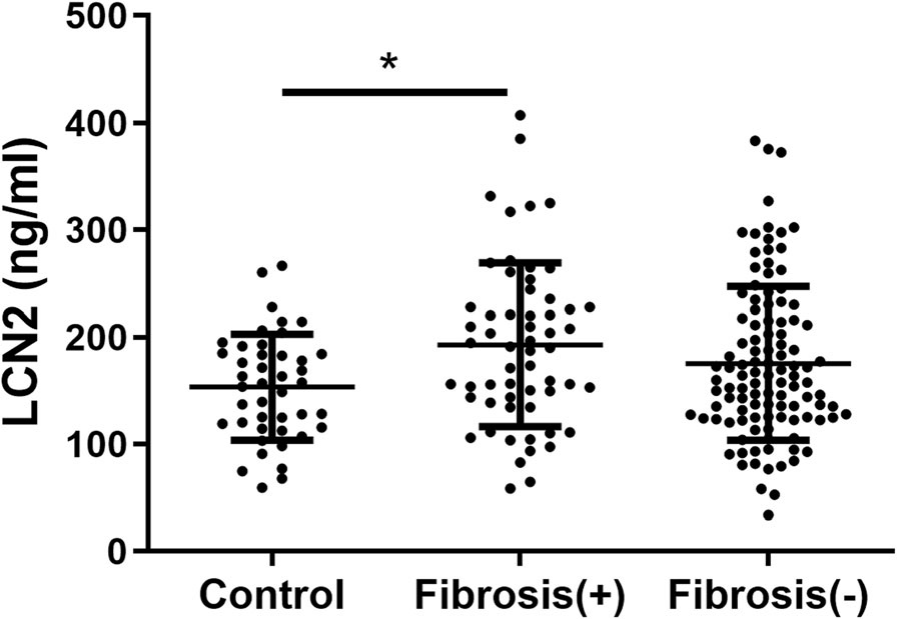
Plasma levels of LCN2 in healthy control, nAMD patients with or without macular fibrosis. Plasma LCN2 were measured by ELISA. Healthy control: *n* = 43; Fibrosis (+): *n* = 58; Fibrosis (−): *n* = 110. * *P* = 0.033, univariate analysis corrected for age

Out of the 174 nAMD participants, 58 had macular fibrosis and 110 did not have fibrosis and 6 were unknown. One-way ANOVA showed a significant difference in the plasma levels of LCN2 among the three groups (controls, nAMD with fibrosis, nAMD without fibrosis, *p* = 0.048), with the highest levels detected in patients with macular fibrosis (192.77 ± 76.37 ng/ml, Fig. 3). Post hoc Tukey test suggested that the difference resided between the groups of controls and nAMD with fibrosis (*p* = 0.037, Fig. 3). The mean age of the control group (73.4 ± 8.9) was significantly younger than that of fibrosis group (79 ± 9.2) and non-fibrosis group (77.9 ± 8.1) (*p* = 0.002, and 0.013 respectively). After adjusting for age, the difference between nAMD with macular fibrosis and controls remains significant (*p* = 0.033, Fig. 3).

### LCN2 and responsiveness to anti-VEGF therapy or Geographic Atrophy (GA)

Out of the 174 nAMD patients, 80 (45.98%) patients responded completely, 90 (51.72%) responded partially, and 4 (2.30%) did not respond to the anti-VEGF therapy. Due to the limited number of non-responders in this study, this group was not included in the statistical analysis. There was no significant difference in the plasma levels of LCN2 between controls (154.21 ± 49.74 ng/ml) and partial responders (178.82 ± 72.35 ng/ml) or complete responders (183.90 ± 75.44 ng/ml). In addition, the plasma levels of LCN2 between patients with GA (181.52 ± 73.03 ng/ml, *n* = 80) and those without GA (178.74 ± 68.69 ng/ml, *n* = 34) were comparable.

## Discussion

LCN2 is known to be produced predominately by neutrophils in the immune system [27]. Previously, we and others have shown that nAMD patients had a higher percentage of circulating neutrophils compared to healthy controls [18, 19, 25, 41]. In this study, we observed a positive correlation between plasma levels of LCN2 and circulating neutrophils in nAMD patients but not in healthy controls (Fig. 2c-d). Our results suggest that higher plasma levels of LCN2 in nAMD patients may, at least in part, attribute to circulating neutrophils. Further studies will be needed to understand the functional difference between neutrophils from nAMD and healthy controls and whether LCN2 is produced or upregulated by a specific subset of neutrophils in nAMD. Interestingly, we also observed a positive correlation between plasma levels of LCN2 and age in nAMD patients but not in controls (Fig. 1a), and there was no correlation between neutrophil and age (Fig. 2d-e), indicating that ageing may affect plasma levels of LCN2 in nAMD independent of circulating neutrophils. Together, our results suggest nAMD patients have abnormal ageing in terms of LCN2 production.

In addition to neutrophils, LCN2 can also be produced by various tissue cells particularly under disease conditions. Plasma and urine levels of LCN2 have been used as a biomarker for kidney disease [42, 43] and cancer progression [44]. Recent studies have shown that retinal cells including Müller glia [45–47], microglia and RPE [47, 48] can all express LCN2 and the expression is upregulated under stress conditions [45, 47, 49]. Furthermore, increased plasma levels of LCN2 have been observed in patients with Stargardt disease, retinitis pigmentosa, and AMD [46]. The diseased macula in nAMD may release LCN2 to the blood circulation contributing to higher plasma levels of this protein.

In this study, the plasma levels of free LCN2 and MMP9/LCN2 complex were comparable in nAMD patients and MMP9 was not significantly increased in nAMD, suggesting that the free form of LCN2, but not the MMP9/LCN2 complex or MMP9 is involved in nAMD. The role of LCN2 in retinal diseases remains controversial. It has been reported that LCN2 participates in light-induced photoreceptor death [50] and the development of early AMD [51]. However, other studies have shown that LCN2 is also involved in retinal pro-survival and anti-oxidative responses [46] and can suppress inflammation and reduce retinal degeneration [45, 46]. In this study, we observed a significantly higher plasma level of LCN2 in nAMD patients with macular fibrosis, suggesting its potential pro-fibrotic roles. LCN2 is reported to contribute to cardiovascular [32] and kidney [31, 52] fibrosis and promote epithelial-to-mesenchymal transition (EMT) in breast cancers [39]. LCN2 is also known to have anti-fibrotic roles as overexpression of LCN2 reduced kidney [34] and liver [33] fibrosis and suppressed epidermal growth factor (EGF) or transforming growth factor-beta (TGFβ)-induced EMT [53]. In addition, LCN2 can bind and stabilise MMP9, which is critically involved in extracellular matrix remodelling during inflammation and wound healing. The role of LCN2 in macular fibrosis warrants further investigation.

The strengths of this study include independent grading of nAMD subtypes, fibrosis, GA and anti-VEGF responsiveness, and extensive exploration of changes in the plasma levels of LCN2, MMP9 and LCN2/MMP9 complex in different types of nAMD as well as in patients partially or completely responding to anti-VEGF treatment. Limitations of the current study include relatively small sample size particularly in subgroups of patients (i.e. RAP *n* = 32, PCV *n* = 23), the recruitment of all participants from one location, and uncontrolled age in control and nAMD groups. In addition, participants were recruited to the study at different times after diagnosis of nAMD, and some patients enrolled into the study at the early stages of nAMD and classified as having no fibrosis or no GA might still develop fibrosis or GA at later time points.

## Conclusions

We demonstrated that the plasma level of LCN2 was positively correlated with age and the percentage of circulating neutrophils in nAMD patients. Higher plasma levels of LCN2 may contribute to the development of macular fibrosis.

## Methods

### Study participants

The study protocol was approved by the Research Ethics Committee of Queen’s University Belfast and procedures were followed the tenets of the Declaration of Helsinki on research into human volunteers. Recruitment criteria for participants include: 1) older than 50 years old, 2) don’t have systemic inflammatory or autoimmune disorders (for examples with active chronic bronchitis, rheumatoid arthritis); 3) are not undergoing steroid therapy or chemotherapy. The nAMD participants were recruited from patients who attended the Macular disease clinics in Belfast Health and Social Care Trust, UK. The healthy controls were accompanying persons of the nAMD patients, who were either spouses, relatives or friends. Written informed consent was obtained from every participant. Participant’s information was ascertained using a structured questionnaire including medical history, current medication, family history of AMD, smoking habits (current, ex-smoker and never-smoker) and body mass index (BMI).

The eye conditions of healthy controls were confirmed by clinical examination via fundus photography and spectral domain-optical coherence tomography (SD-OCT). nAMD were diagnosed by clinical examination of colour fundus photography (CFP), autofluorescence, SD-OCT, fluorescein angiography and indocyanine green angiography. nAMD subgroups including CNV, RAP and PCV were identified at the time of recruitment. Participants with nAMD received intravitreal anti-VEGF treatment (Lucentis) and were followed up for over 6 months. Their fundus colour images and tomographic scans were graded at baseline and their most recent hospital visit prior to closure of the database. Responsiveness to treatment was defined based on the participant achieving a fluid-free macula at any stage during follow-up in the treated eye. In addition, the status of whether a patient was fluid free at the month-3 and month-6 examinations was also recorded. Participants were classified into the following three categories: (1) complete responder: resolution of leakage at any time point during follow up; (2) partial responder: exhibiting dependence on VEGF inhibitors but a fluid-free macula never achieved; and (3) non-responder: no morphological improvement or worsening. Macular scar identification was based on both CFP and OCT characteristics. On CFP, macular scar was defined as well-delineated areas of yellowish-white tissue, which on OCT corresponded to the presence of linear bands of hyper reflective material that had either obscured or replaced the normal hyper reflective bands of the neurosensory retina and RPE/Bruch’s membrane complex. Macular atrophy (MA) was defined as single or multiple areas of hypopigmentation with well-defined borders and visible large choroidal vessels on CFP, which corresponded to window defects on angiography and/ or to the loss of cellular layers (outer retina, RPE and choriocapillaris) on the accompanying tomograms [12, 19, 20].

### Sample collection

Peripheral blood samples were drawn into EDTA treated tubes and transferred to the research lab at room temperature. Plasma was extracted within 3 h after blood collection. Plasma was first separated from the whole blood with centrifugation of 10 min at 300 g and followed by further centrifugation of 10 min at 2000 g to remove cell debris or platelets. Plasma aliquots were stored at -80 °C until analysis.

### Flow cytometry analysis of neutrophils

Circulating neutrophils were identified by flow cytometry using the protocol described in our previous publications [19, 54]. In brief, 30 μl of fresh blood were incubated with fluorochrome-labelled antibody cocktail (CD19-FITC, CD16-Pacific Blue, CD11b-APC (all from BD Biosciences, Oxford, UK) and HLA-DR-PE (eBioscience, UK)) for 45 min in the dark at 4 °C. Red blood cells were removed with lysis buffer (BD Biosciences) and samples were acquired using a BD Canto II flow cytometer (BD Biosciences). The flow cytometry data were analysed using FlowJo (version 10, Tree Star Inc., Ashland, OR, USA). Neutrophils were identified based on their cell size (FSC) and granularity (SSC) as well as their cell surface antigens (CD11b^+^CD16^+^HLA-DR^−^).

### Enzyme-Linked Immunosorbent Assay (ELISA)

LCN2, MMP9 and MMP9/LCN2 complex DuoSet ELISA kits were purchased from R & D systems (Abingdon, Oxford, UK). The ELISAs were performed following the protocols provided by the manufacture. The results were measured using a plate reader at the wavelength of 450 nm and correction at 560 nm. A standard curve was generated using GraphPad following a four-parameter logistic (4-PL) curve-fit.

### Statistical analysis

Statistical analysis was performed using the Statistical Package for the Social Sciences (SPSS), window version 25 (the International Business Machines Corporation, IBM). All continuous variables were firstly tested for normality using the Kolmogorov-Smirnov test. Independent-Samples t-test or One-way ANOVA were used to analyse normally distributed continuous variables; whereas nonparametric test including Mann-Whitney U test (for two samples) and Kruskal-Wallis one-way ANOVA (for more than two samples) were used to examine continuous variables which were not normally distributed. Pearson’s Chi-square test was used to examine categorical variables such as demographic information and clinical data. Bivariate correlation was performed using Pearson’s correlation. Univariate analysis was performed to examine associations of variables. Data were presented as mean ± SD, *p* value < 0.05 was considered as statistically significant in all cases.

## Data Availability

The datasets generated and analysed during the current study are available from the corresponding author on reasonable request.

## Abbreviations

AMD: Age-related macular degeneration
BMI: Body mass index
CNV: Choroidal neovascularisation
EMT: Epithelial-to-mesenchymal transition
nAMD: Neovascular age-related macular degeneration
GA: Geographic atrophy
LCN2: Lipocalin-2
MMP9: Matrix metalloproteinase 9
PCV: Polypoidal choroidal vasculopathy
RAP: Retinal angiomatous proliferation
RPE: Retinal pigment epithelium
SD-OCT: Spectral domain optical coherence tomography
VEGF: Vascular endothelial growth factor
